# How attention and reinforcers jointly optimize the associations between sensory representations, working memory and motor programs

**DOI:** 10.1186/1471-2202-12-S1-P267

**Published:** 2011-07-18

**Authors:** Jaldert O Rombouts, Sander M Bohte, Pieter R Roelfsema

**Affiliations:** 1 Life Sciences, Centrum Wiskunde & Informatica, Amsterdam, 1098 XG, The Netherlands; 2 The Netherlands Institute for Neurosciences, Royal Netherlands Academy of Arts and Sciences, Amsterdam, 1105 BA, The Netherlands; 3 Department of Integrative Neurophysiology, Free University, Amsterdam, 1081 HV, The Netherlands

## 

Almost all animal behaviors can be seen as sequences of actions towards achieving certain goals. How the association cortices learn to link sensory stimuli to a correct sequence of motor responses is not well understood, especially when only a correct sequence of responses is rewarding.

We present a biologically plausible neuronal network model that can be trained to perform a large variety of tasks when only stimuli and reward contingencies are varied. The model’s aim is to learn action values in a feedforward neuronal network and we present mechanisms to overcome the structural and temporal credit assignment problems. The temporal credit assignment problem is solved by a form of Q-learning [1]. The structural credit assignment problem is solved by a form of ‘attentional’ feedback from motor cortex to association cortex that delineates the units that should change connectivity to improve behavior [2]. Moreover, the model has a new mechanism to store traces of relevant sensory stimuli in working memory.

During learning, the sensory stimuli, in combination with traces of previous stimuli in working memory become associated with a unique set of action values. Learning in the model is biologically realistic as model units have Hebbian plasticity that is gated by two factors [2]. Firstly, reinforcers or increases in reward expectancy cause the global release of neuromodulatory signals that inform all synapses of the network if the outcome of a trial was better or worse than expected [3]. Selective attention is the second factor that gates plasticity. Attentional feedback highlights the chain of neurons between sensory and motor cortex responsible for the selected action. Only neurons that are causally linked to the action receive attentional feedback, and change the strength of their connections. Selective attention thereby solves the structural credit assignment problem. The resulting learning rule is a form of AGREL [2], which was previously shown to be on average equivalent to error-backpropagation in classification tasks with immediate reward. The present generalization of the learning scheme is based on temporal difference learning and it can train multilayer feedforward networks to perform delayed reward tasks with multiple epochs that require multiple behavioral responses. Importantly, the generalization *MQ-AGREL* learns to store in working memory information that is relevant at a later stage during a task. This memory is maintained by persistent activity of units at the intermediate network layers. We show that MQ-AGREL can be trained in many tasks that are in use in neurophysiology, including (1) (delayed) saccade-antisaccade tasks; (2) categorization tasks; and (3) probabilistic classification tasks.

Neurons at intermediate levels of the network acquire visual responses and memory responses as the result of training that resemble the tuning of neurons in association areas of the cerebral cortex of animals that are trained in these same tasks. We conclude that MQ-AGREL is a powerful and biologically realistic learning rule that accounts for learning in delayed reward tasks that involve non-linear mappings from sensory stimuli and working memory onto motor responses.
